# Gatifloxacin hydrochloride confers broad-spectrum antibacterial activity against phytopathogenic bacteria

**DOI:** 10.3389/fmicb.2024.1504243

**Published:** 2024-12-02

**Authors:** Yanxia Huang, Bin Peng, Chenhui Li, Yuqin Wu, Zixian Zeng, Moh Tariq, Lin Jiang, Shun-xiang Li, Dousheng Wu

**Affiliations:** ^1^Hunan Key Laboratory of Plant Functional Genomics and Developmental Regulation, College of Biology, Hunan University, Changsha, China; ^2^Hunan Engineering Technology Research Center for Bioactive Substance Discovery of Chinese Medicine, School of Pharmacy, Hunan University of Chinese Medicine, Changsha, China

**Keywords:** gatifloxacin hydrochloride, antibacterial, *Ralstonia solanacearum*, *Pseudomonas syringae*, *Xanthomonas campestris* pv. Vesicatoria

## Abstract

Bacterial diseases pose significant threats to agriculture and natural ecosystems, causing substantial crop losses and impacting food security. Until now, there has been a less efficient control strategy against some bacterial diseases such as bacterial wilt, caused by *Ralstonia solanacearum*. In this study, we screened a library of 57 microorganism-derived small molecule compounds and 1 fully synthetic small molecule compound for their antibacterial activity against *R. solanacearum*. Gatifloxacin hydrochloride exhibited the best inhibitory effect with an inhibition rate of 95% at 0.0625 mg/L. Further experiments demonstrate that gatifloxacin hydrochloride inhibits *R. solanacearum* growth in a concentration-dependent manner, with the minimum inhibitory concentration of 0.125 mg/L. Treatment with 0.5 mg/L of gatifloxacin hydrochloride killed more than 95% of bacteria. Gatifloxacin hydrochloride significantly inhibited biofilm formation by *R. solanacearum*. Gatifloxacin hydrochloride also shows good antibacterial activity against *Pseudomonas syringae* pv. *tomato* DC3000 and *Xanthomonas campestris* pv. *vesicatoria*. Exogenous application of gatifloxacin hydrochloride suppressed disease development caused by *R. solanacearum* and *P. syringae*. In summary, our results demonstrate the great potential of microorganism-derived and synthetic small molecules as broad-spectrum antibacterial compounds, providing alternative ways for the efficient control of bacterial plant diseases.

## Introduction

Plant diseases caused by bacterial pathogens pose a significant threat to agriculture, causing substantial yield losses and affecting food security (Singh et al., 2023). Bacterial pathogens can infect various plant tissues, leading to symptoms such as wilting, necrosis, chlorosis, and rot (Nazarov et al., 2020; Wang et al., 2022). The bacteria can spread through air, water, or vectors such as insects and can survive in the soil or on plant debris for extended periods (Voegele and Mendgen, 2011; Trivedi et al., 2020; Ahmed et al., 2022). Based on current understanding, bacterial pathogens from more than 25 genera and over 200 species are pathogenic on different plant species (Buttimer et al., 2017). Mansfield et al. constructed a list of the top 10 bacterial plant pathogens according to their scientific and economic importance (Mansfield et al., 2012). *Pseudomonas syringae,* which includes many pathovars and causes severe plant diseases such as bacterial speck of tomato, is ranked as the most important bacterial pathogen. The second important bacterium is *Ralstonia solanacearum*, the causal agent of bacterial wilt. Other important bacteria include *Agrobacterium tumefaciens*, *Xanthomonas* species, and *Erwinia amylovora* (Mansfield et al., 2012; Timilsina et al., 2020).

*Ralstonia solanacearum* is a soil-borne bacterium known for causing bacterial wilt in a wide range of host plants, including many of economic importance such as tomatoes, potatoes, peppers, eggplants, and tobacco (Vailleau and Genin, 2023). First identified in the late 19th century, this pathogen has since become one of the most devastating plant diseases worldwide (Ahmed et al., 2022). It is responsible for significant yield losses, with some reports indicating that it infects over 450 plant species (An and Zhang, 2024). The bacterium survives in the soil for extended periods, and its spread is linked to the movement of plant products and propagation materials (Caldwell et al., 2017). The life cycle of a pathogen involves chemotactic movement toward plant roots, where it infects through wounds or natural openings (Ahmed et al., 2022). Once inside, it multiplies rapidly in the xylem vessels and produces extracellular polysaccharides that obstruct the water-conducting tissues of the plant, leading to wilting and eventual plant death (Lowe-Power et al., 2018). Control measures for *R. solanacearum* include cultural practices, biological control using beneficial microbes, and the use of resistant crop varieties (Yuliar et al., 2015; Ahmed et al., 2022; Vailleau and Genin, 2023). However, effective and environmentally friendly control measures are still lacking.

A range of strategies, including agricultural practices, biological controls, and chemical treatments, are typically deployed to control bacterial plant diseases (Collinge et al., 2022). Resistance breeding is also encouraged to combat bacterial disease where possible (Wang et al., 2018). However, due to the big variation in pathogen infection and disease development, the accessibility of these management options is subject to the particular crop and pathogen involved (Nelson et al., 2018; Singh et al., 2023). Moreover, certain technological solutions might not align with the agricultural production systems or comply with the existing regulatory frameworks (Benyam et al., 2021; Brunelle et al., 2024). For example, agricultural practices such as crop sanitation and soil disinfection can be effective in curbing the incidence of diseases and preventing their spread, but they often need to be complemented with other methods for adequate control (Lodovica Gullino et al., 2022). Therefore, the management of bacterial disease is still challenging. Another significant problem in disease management is the evolution of bacterial pathogens, which can rapidly develop resistance to certain control measures (Trivedi et al., 2020; Larsson and Flach, 2022; Ahmed et al., 2024). This necessitates continuous research and development of new strategies. In addition, the global trade of plants and seeds can inadvertently spread bacterial pathogens to new regions, complicating disease management efforts (Tsykun et al., 2022; Singh et al., 2023).

Using natural products to control plant bacterial diseases is a vibrant field with significant potential (Chopra and Dhingra, 2021; Li et al., 2024). Natural compounds derived from various sources, including plants, bacteria, and fungi, have demonstrated antagonistic activity against a range of plant pathogens (Álvarez-Martínez et al., 2021; Matrose et al., 2021; Li et al., 2024). These natural substances can be extracted and purified using established and emerging methods, and they hold promise as alternatives to synthetic pesticides, which can have negative impacts on the environment and human health (Atanasov et al., 2021; Shetty et al., 2023; Ahmad et al., 2024). Advancements in the discovery of plant-derived antimicrobial compounds have been made in recent years, with a focus on combating antimicrobial resistance in pathogens (Woo et al., 2023; Li et al., 2024). Many alkaloids, phenols, and terpenes have shown activity against plant-pathogenic bacteria (Woo et al., 2023). For instance, several plant-derived antimicrobial compounds, including new 8-O-4′ neolignans, harmine, and coumarins, have been shown to have good antibacterial activity against *R. solanacearum* (He et al., 2020; Yang et al., 2021; Xia et al., 2023). However, less is known about the potential of microorganism-derived compounds in controlling bacterial plant diseases.

Gatifloxacin hydrochloride is a fully synthetic small molecule with promising antibacterial activities against both Gram positive and Gram-negative bacteria, including some anaerobic organisms and mycobacteria (Perry et al., 2002). Its primary mechanism of action is the inhibition of bacterial enzymes topoisomerase II (DNA gyrase) and topoisomerase IV, which are essential for bacterial DNA replication, transcription, repair, and recombination. This inhibition leads to the prevention of bacterial cell division and ultimately results in bacterial cell death (Rafii et al., 2005). Because of its good antibacterial activity, gatifloxacin is indicated for the treatment of various infections. For example, gatifloxacin ophthalmic solution is clinically used for the treatment of bacterial conjunctivitis, a common infectious disease of the eye (Cervantes and Mah, 2011). In addition to being used separately, gatifloxacin has been combined with copper and zinc, which show similar or better antibacterial activity against the microorganisms *X. campestris*, *Escherichia coli*, *Bacillus subtilis,* and *Staphylococcus aureus* (Kostelidou et al., 2016; Kakoulidou et al., 2019). Despite its broad antibacterial activity on human and animal bacterial pathogens, less is known about the bioactivity of gatifloxacin on plant pathogenic bacteria.

In this study, we screened a compound library comprising 57 microorganism-derived compounds and 1 fully synthetic compound to identify compounds that show good antibacterial activity against *R. solanacearum*. We found that gatifloxacin hydrochloride can be used as a promising antibacterial agent against *R. solanacearum*. Furthermore, gatifloxacin hydrochloride also shows antibacterial activity against *P. syringae* and *Xanthomonas campestris* pv. *vesicatoria*. Application of gatifloxacin hydrochloride effectively delayed disease development caused by *R. solanacearum* and *P. syringae*.

## Materials and methods

### Bacterial strains and growth conditions

The *R. solanacearum* strain GMI1000 was grown in B liquid medium (1% peptone, 0.1% tryptone, 0.1% yeast extract, and 2.5% glucose) or B solid medium supplemented with 1.5% agar at 28°C. The *P. syringae* and *X. campestris* pv. *vesicatoria* were grown in LB liquid medium (1% peptone, 0.5% yeast extract, and 1% NaCl) containing rifampicin (0.005%) or LB solid medium supplemented with 1.5% agar and rifampicin (0.005%) at 28°C.

### Small molecule compounds information

In this study, all small molecule compounds (HPLC purity >99%) were acquired from TargetMol (Shanghai, China, Supplementary Table S1). These compounds were dissolved in dimethyl sulfoxide (DMSO) to achieve final concentrations of 1 mM or as indicated and subsequently stored at −20°*C*. Prior to experimentation, the frozen compound solutions were thawed at room temperature for 10 min and then incorporated into the B or B agar media at the designed concentrations. The equivalent volume of DMSO served as a solvent control.

### Screening of bioactive antibacterial compounds

Antibacterial activity screening of compounds was conducted utilizing 1.5-mL test tubes. Specifically, 282 μL of B liquid culture medium was dispensed into each tube, followed by the addition of 15 μL of bacterial suspension of OD_600_ = 0.01. Subsequently, 3 μL of 1 mM compound solution was incorporated to achieve a final concentration of 10 μM. An equivalent volume of DMSO served as a control treatment. The resulting mixtures were incubated at 28°C on a shaking incubator set with 180 rpm for 24 h, after which the OD_600_ values were measured. Each treatment was performed in triplicate to ensure the reliability of the results.

### Determination of the bacterial growth curve

The growth curve of *R. solanacearum*, *P. syringae*, or *X. campestris* pv. *vesicatoria* was performed as previously described with minor modifications (Xia et al., 2023). In brief, in a 5 mL B or LB liquid medium, 25 μL of a bacterial suspension (OD_600_ = 0.1) was inoculated. Subsequently, varying concentrations of gatifloxacin hydrochloride were added to achieve final concentrations of 0.0156 mg/L, 0.03125 mg/L, 0.0625 mg/L, 0.125 mg/L, and 0.25 mg/L. An equivalent volume of DMSO was utilized as the solvent control group. The mixed culture was incubated at 28°C with continuous shaking at 180 rpm for 24 or 36 h, after which the OD_600_ was measured at 2-or 3-h intervals. Each treatment was conducted in triplicate to ensure statistical reliability and reproducibility of the results.

### Determination of bactericidal activity

The evaluation of bactericidal activity was carried out as previously described with minor modifications (Xia et al., 2023). In brief, one single colony of *R. solanacearum*, *P. syringae*, or *X. campestris* pv. *vesicatoria* was selected from the B or LB agar plate and cultured overnight in a B or LB liquid medium. The following day, the bacterial suspension was subjected to centrifugation to remove the supernatant, and the resulting pellet was resuspended under sterile conditions. The OD_600_ of the suspension was adjusted to 0.1. The bacterial suspension was then aliquoted into 15-mL centrifuge tubes, each containing 3 mL of the suspension. Gatifloxacin hydrochloride was subsequently added to the tubes to achieve final concentrations of 0.25 mg/L, 0.5 mg/L, 1 mg/L, 2 mg/L, or 4 mg/L. An equal volume of DMSO was included as a solvent control. The treated bacterial suspensions were incubated in a shaking incubator at 180 rpm and 28°C. After incubation for either 1 or 2 h, 100 μL of the suspension was diluted in sterile water to create serial dilutions. Following dilution, 5 μL of each dilution was plated onto the B agar plates. The plates were incubated at 28°C for 48 h. After the incubation period, colonies were enumerated to assess the viability of bacteria in each treatment group. The bactericidal efficacy was calculated based on the colony counts.

### Biofilm formation analysis

The analysis of biofilm formation was conducted following the methodology established by Wu et al. (2015). In a 96-well polystyrene microplate, 190 μL of liquid B medium and 10 μL of *R. solanacearum* suspension adjusted to OD_600_ = 0.1 were added to each well. Subsequently, gatifloxacin hydrochloride was introduced to each well to achieve final compound concentrations of 0.0625 mg/L, 0.125 mg/L, or 0.25 mg/L, while an equivalent volume of DMSO was used as a negative control. The 96-well plate containing the mixture was statically incubated at 28°C for a duration of 24 h. The culture medium was carefully aspirated using a micropipette, and each well was rinsed gently with 200 μL of sterile water to remove any residual medium. Following this, 220 μL of 0.1% crystal violet solution was added, and the wells were stained at room temperature for 30 min. Upon completion of the staining procedure, the crystal violet solution was aspirated, and each well was washed two times with 200 μL of sterile water to eliminate any remaining dye. Subsequently, the 96-well plate was placed in a fume hood to air-dry for 30 min. After drying, 200 μL of 95% ethanol was added to each well to solubilize the crystal violet that had adhered to the biofilm matrix. Finally, the optical density of the liquid in each well was measured at 530 nm using a microplate reader, providing quantitative data on biofilm formation.

### Inoculation assay

The efficacy of gatifloxacin hydrochloride in controlling bacterial wilt in tomatoes was assessed using a naturalistic soil-soaking method. A 10-mL solution of 4 mg/L gatifloxacin hydrochloride was irrigated to the roots of 4-week-old tomato plants, with an equivalent volume of DMSO serving as a negative control. After 24 h, a 10 mL suspension of *R. solanacearum* (OD_600_ = 0.1) was inoculated. The inoculated plants were maintained in a greenhouse at 28°C under a light cycle of 14 h in light and 10 h in dark conditions. Symptoms of wilting were recorded daily. Disease severity was evaluated using a scoring system ranging from 0 to 4: 0 indicated no wilting symptoms; 1 represented 1 to 25% leaf wilt; 2 indicated 26 to 50% leaf wilt; 3 represented 51 to 75% leaf wilt; and 4 indicated 76 to 100% leaf wilt.

### Antimicrobial activity determination of *Pseudomonas syringae* and *Xanthomonas campestris* pv*. vesicatoria*

In brief, 25 μL of a suspension of *P. syringae* and *X. campestris* pv. *vesicatoria* at an OD_600_ of 0.1 was added to 5 mL of LB liquid medium, followed by the addition of different amounts of gatifloxacin hydrochloride to achieve final concentrations of 0.015 mg/L and 0.5 mg/L, respectively. An equal volume of DMSO was used as a negative control. After mixing, the liquid was incubated for 24 h at 28°C with shaking at 180 rpm. The OD_600_ values of the bacterial suspensions in different treatment groups were measured. Each treatment was performed with three technical replicates.

### Inoculation analysis of *Pseudomonas syringae*

Overnight cultures of *P. syringae* were subjected to centrifugation and subsequently resuspended in a 10 mM MgCl₂ solution, with the OD_600_ of the resuspended culture adjusted to 0.1. This bacterial suspension was uniformly applied to the foliage of 4-week-old tomato plants, followed by the administration of a working concentration of 4 mg/L of gatifloxacin hydrochloride solution. A corresponding volume of DMSO dilution was employed as the control treatment. The inoculated plants were maintained in a growth chamber at 28°C, with 70% relative humidity and a photoperiod of 14-h light and 10-h dark. Phenotypic assessments of the inoculated leaves were conducted 8 and 10 days post-inoculation. For each treatment, three leaf disks measuring 0.5 cm^2^ were collected and homogenized in 1 mL of sterile water. A 100 μL aliquot of this homogenate was subsequently diluted in sterile water to generate serial dilutions. Each diluted suspension was plated in 10 μL volumes onto the LB agar plates containing rifampicin. Following a 48-h incubation at 28°C, bacterial titers for each treatment were quantified, ensuring a minimum of six replicates per experimental group.

### Statistical analysis

The data were analyzed with either Excel or GraphPad Prism using Student’s *t*-test (*p < 0.05, **p < 0.01, ***p < 0.001, and ****p < 0.0001) or one-way analysis of variance (ANOVA) for multiple comparisons (*p* < 0.05).

## Results

### A screen identifies gatifloxacin hydrochloride as a novel antibacterial agent against *Ralstonia solanacearum*

**Figure 1:**
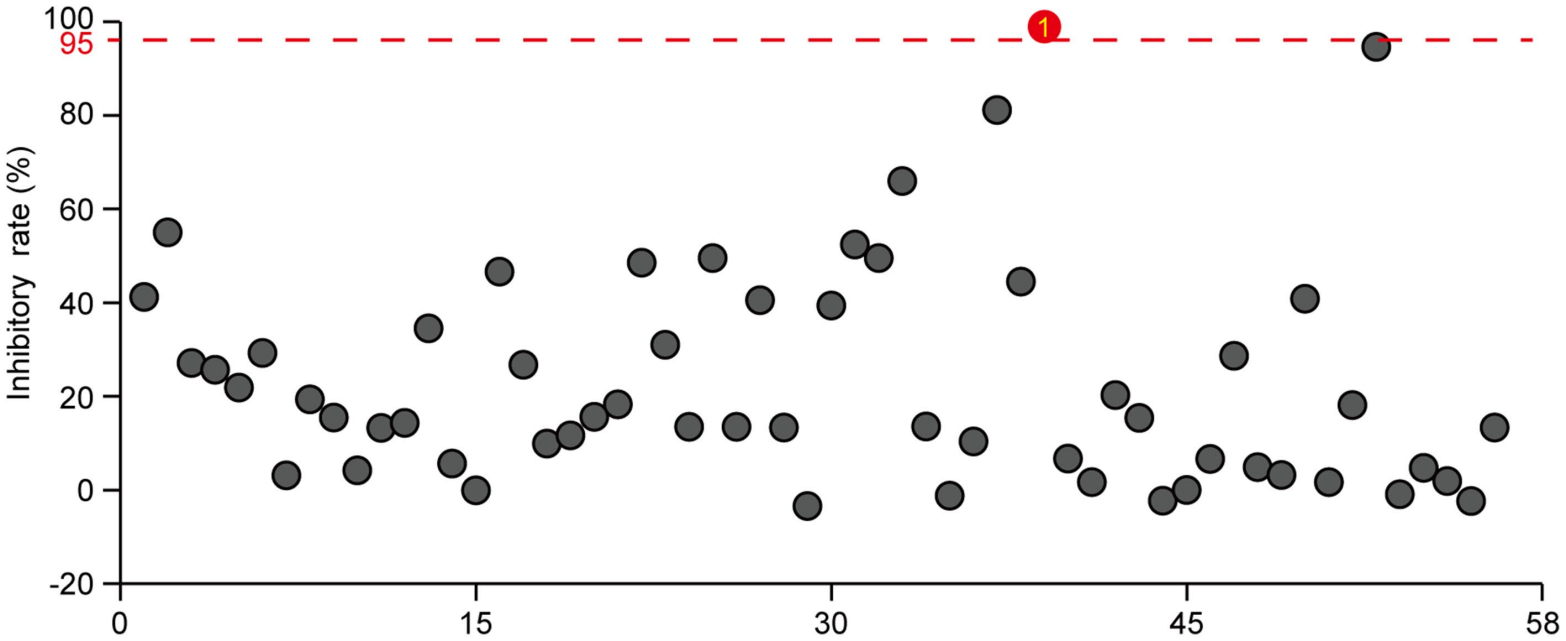
Screening of microorganism-derived and synthetic compounds against *R. solanacearum*. The effects of 57 microorganism-derived small molecules and 1 fully synthetic compound on the growth of *R. solanacearum* were investigated. DMSO served as the negative control, with a threshold of 95% bactericidal activity within 24 h as the criterion for identifying effective bactericides against *R. solanacearum*. The reported values are means (*n* = 3). For the natural products exhibiting bactericidal rates exceeding 95%, three independent experiments were conducted, all yielding consistent results.

We previously screened a plant-derived compound library and identified harmine as an antibacterial agent against *R. solanacearum*, with a minimum inhibitory concentration of 120 mg/L (Xia et al., 2023). To further identify natural compounds that can inhibit *R. solanacearum* growth, we screened a library consisting of 57 microorganism-derived small molecule compounds and 1 fully synthetic small molecule compound and tested their antibacterial activity against *R. solanacearum* at a concentration of 10 μM. Many of those compounds inhibited the growth of *R. solanacearum*, with the inhibitory rate varying from 40 to 97% (Figure 1; Supplementary Table S2). Among those bioactive compounds, four compounds, namely, gatifloxacin hydrochloride, paromomycin sulfate, fervenulin, and 2,3-butanediol, exhibited a very strong inhibitory effect on the growth of *R. solanacearum* in a liquid medium, with an inhibitory rate higher than 60% (Figure 1). Gatifloxacin hydrochloride showed the best inhibitory effect, with an inhibitory effect rate of 96%. Other compounds, such as indoleacetic acid and 3-methyluridine, also exhibited an inhibitory effect on the growth of *R. solanacearum*, though the inhibitory effect was not as strong as that of the above-mentioned four compounds. In summary, the screening identified several small molecule compounds as novel antibacterial agents against *R. solanacearum*.

**Figure 2:**
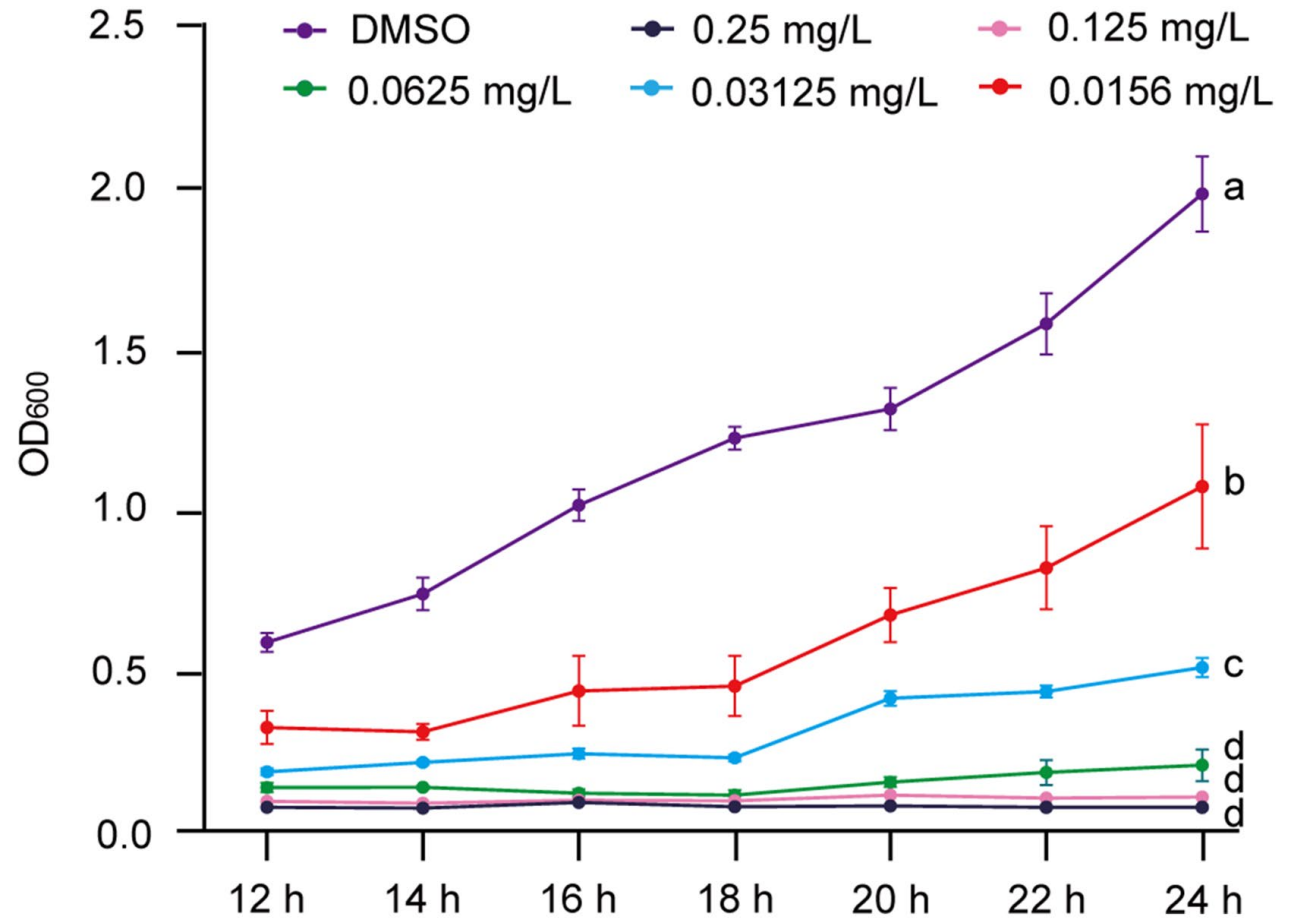
Gatifloxacin hydrochloride exhibits a concentration-dependent inhibition of *R. solanacearum* growth. The growth curve of *R. solanacearum* was determined in B liquid medium supplemented with varying concentrations of gatifloxacin hydrochloride and DMSO. Following a 12-h co-incubation period with different concentrations of gatifloxacin hydrochloride or DMSO, the OD_600_ was measured, with subsequent measurements taken every 2 h. The minimum inhibitory concentration (MIC) was defined as the lowest concentration at which the OD_600_ value of the mixture remained below 0.1 after 24 h of co-culturing. Data are presented as mean ± standard deviation (*n* = 3). Different lowercase letters indicate statistical significance (one-way ANOVA, *p* < 0.05).

To further explore the inhibitory effect of gatifloxacin hydrochloride on *R. solanacearum* growth, we assessed its effectiveness across a concentration range of 0.0156 mg/L to 0.25 mg/L. The growth curves from 12 to 24 h in liquid culture revealed that all tested concentrations significantly inhibited the growth of *R. solanacearum* (Figure 2). In particular, growth was almost completely suppressed at concentrations of 0.25 mg/L and 0.125 mg/L. The inhibitory effect decreased as the concentration of gatifloxacin hydrochloride was lowered, with the inhibition rate dropping to 45% at 0.0156 mg/L.

### Gatifloxacin hydrochloride exhibits bactericidal activity against *Ralstonia solanacearum*

**Figure 3:**
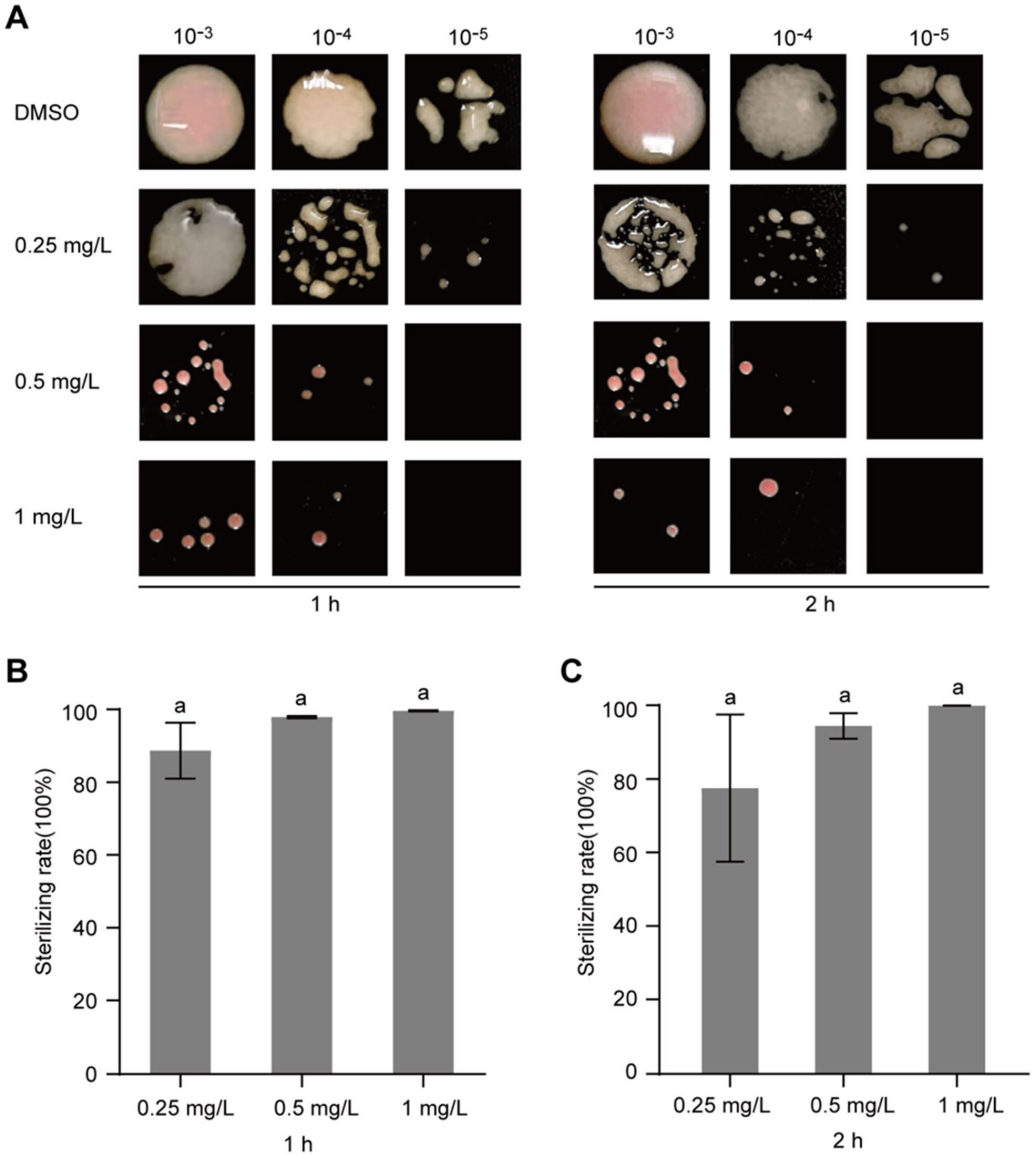
Gatifloxacin hydrochloride exhibits bactericidal activity against *R. solanacearum*. Different concentrations of gatifloxacin hydrochloride and DMSO were added to a suspension of *R. solanacearum* at an OD_600_ of 0.1, followed by co-incubation of the mixture at 28°C. After treatment for either 1 h or 2 h, the mixture was sampled for gradient dilution. The diluted bacterial suspension was plated onto the B agar plates and incubated at 28°C for 48 h. (A) Colony shape of *R. solanacearum* after gatifloxacin hydrochloride or DMSO treatment. (B–C) The number of surviving *R. solanacearum* in the 10^−4^ dilution was quantified, using the bacterial count from the DMSO treatment group as a control to calculate the bactericidal rate. Data are presented as mean ± standard deviation (*n* = 3). Different lowercase letters indicate statistical significance (one-way ANOVA, *p* < 0.05).

After confirming that gatifloxacin hydrochloride inhibits the growth of *R. solanacearum*, we next investigated whether it could exhibit bactericidal activity. To test this, *R. solanacearum* was first cultured in a liquid medium and then treated with various concentrations of gatifloxacin hydrochloride, ranging from 0.25 mg/L to 1 mg/L. Following 1-h and 2-h treatments, the bacterial cells were harvested, resuspended in sterile water, and plated on agar to assess viable bacterial counts. Compared to the DMSO control, treatment with 0.25 mg/L of gatifloxacin hydrochloride resulted in an 89% reduction in bacterial viability at both 1 and 2 h, while 1 mg/L killed 99% of the bacteria (Figure 3). These findings indicate that gatifloxacin hydrochloride exhibits potent bactericidal activity against *R. solanacearum*.

### Gatifloxacin hydrochloride inhibits biofilm formation by *Ralstonia solanacearum*

**Figure 4:**
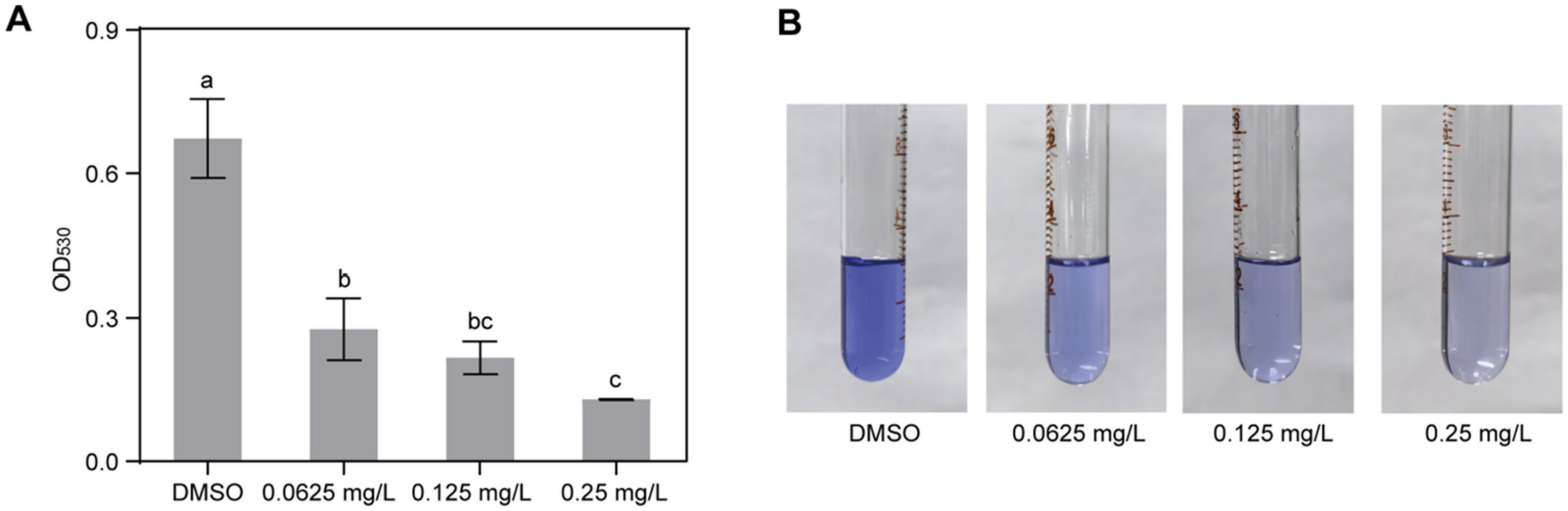
Gatifloxacin hydrochloride inhibits the formation of biofilm by *R. solanacearum*. DMSO and various concentrations of gatifloxacin hydrochloride were added into B liquid medium containing *R. solanacearum* and co-cultured for 24 h, after which biofilm quantification (A) and visualization (B) were performed. The data are presented as mean ± standard deviation (*n* = 3). Different lowercase letters indicate statistical significance (one-way ANOVA, *p* < 0.05).

To assess the impact of gatifloxacin hydrochloride on the biofilm formation of *R. solanacearum*, a standard polyvinyl chloride (PVC) assay was employed. Gatifloxacin hydrochloride was tested at concentrations ranging from 0.0625 mg/L to 0.25 mg/L, and biofilm levels were quantified in its presence and absence. Biofilm formation was notably decreased in all gatifloxacin hydrochloride treatments when compared to the DMSO control. Specifically, gatifloxacin hydrochloride at 0.0625 mg/L reduced biofilm formation by 58% (Figure 4A). With the increase in gatifloxacin hydrochloride concentration, the biofilm levels were lower. Gatifloxacin hydrochloride at 0. 25 mg/L reduced biofilm formation by 80% (Figure 4A). We further visualized the biofilm with crystal violet. Different concentrations of gatifloxacin hydrochloride treatments substantially reduced the purple color compared to the DMSO control, indicating inhibition of biofilm formation by gatifloxacin hydrochloride (Figure 4B). Taken together, these results suggest that gatifloxacin hydrochloride reduces the biofilm formation by *R. solanacearum*.

### Gatifloxacin hydrochloride delays bacterial wilt disease development

**Figure 5:**
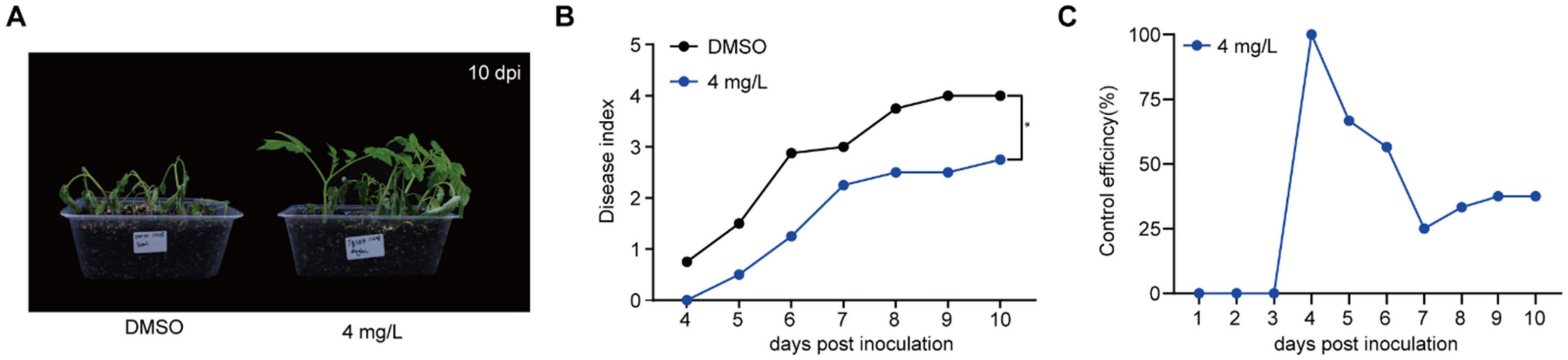
Gatifloxacin hydrochloride significantly delays the progression of bacterial wilt in tomato plants. **(A,B)** Four-week-old tomato seedlings were pretreated with either 10 mL of DMSO or 4 mg/L of gatifloxacin hydrochloride for 24 h, followed by inoculation with 10 mL of *R. solanacearum* suspension (OD_600_ = 0.2). **(A)** The wilting symptom of tomato treated with DMSO or 4 mg/L of gatifloxacin hydrochloride at 10 days post inoculation. **(B)** Wilting symptoms were monitored daily post-inoculation using a disease index scoring system ranging from 0 to 4 (0: no wilting symptoms; 1: 1–25% leaf wilting; 2: 26–50% leaf wilting; 3: 51–75% leaf wilting; 4: 76–100% leaf wilting). Each data point represents the mean disease index from eight plants, with statistical significance noted at *p < 0.05 (Student’s *t*-test). (C) The control efficiency of gatifloxacin hydrochloride on bacterial wilt in tomato plants. The formula for calculating the control efficiency is as follows: Control efficiency % = (Disease index of DMSO-treated plants – Disease index of gatifloxacin hydrochloride-treated plants)/Disease index of DMSO-treated plants × 100.

Given that gatifloxacin hydrochloride shows strong antibacterial activity against *R. solanacearum* and reduces biofilm formation, we hypothesized that this small molecule compound could delay bacterial wilt disease development caused by this pathogen. *R. solanacearum* has a wide range of host plants, with Solanaceae plants, particularly tomato, being the most common hosts (Vailleau and Genin, 2023). We treated 4-week-old tomato plants with either DMSO or 4 mg/L of gatifloxacin hydrochloride, followed by inoculating with *R. solanacearum* using the natural soil drenching method. Compared to DMSO control, gatifloxacin hydrochloride treatment significantly delayed bacterial wilt disease development (Figures 5A,B). At 10 days post-inoculation, the disease index of DMSO-treated plants was 4, while the disease index of gatifloxacin hydrochloride-treated plants was 2.5. We further calculated the control efficiency of gatifloxacin hydrochloride against bacterial wilt. Gatifloxacin hydrochloride treatment led to 37.5% control efficiency in tomatoes (Figure 5C). These results suggest that gatifloxacin hydrochloride has the potential to be an effective antibacterial agent for the control of bacterial wilt caused by *R. solanacearum*.

### Gatifloxacin hydrochloride exhibits broad-spectrum antibacterial activity against *Pseudomonas syringae* and *Xanthomonas campestris* pv. *vesicatoria*

**Figure 6:**
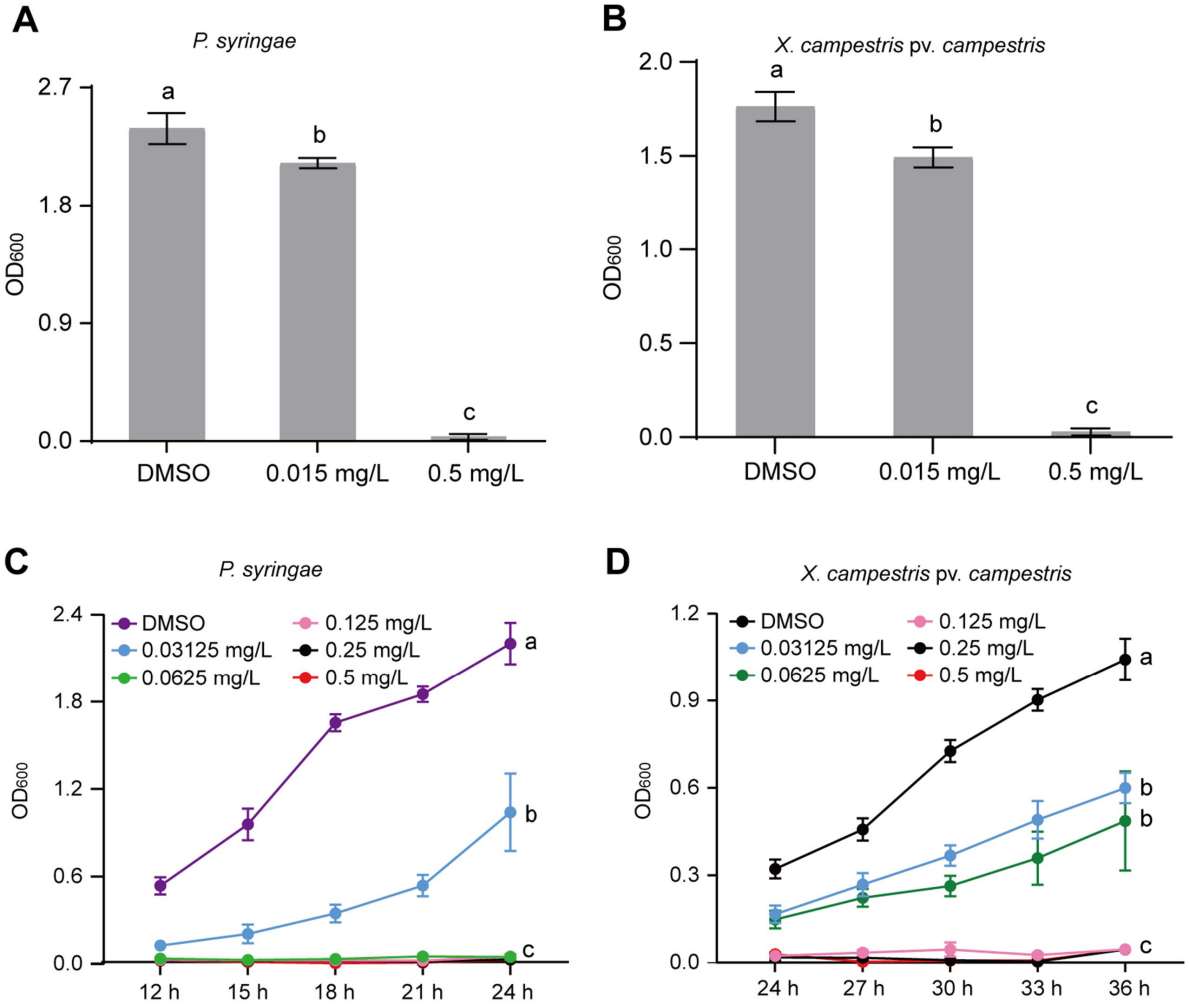
Gatifloxacin hydrochloride has broad-spectrum antimicrobial activity against *P. syringae* and *X. campestris* pv. *vesicatoria*. (A,B) The growth of *P. syringae* and *X. campestris* pv. *vesicatoria* in liquid LB medium after 24-h incubation. Different concentrations of gatifloxacin hydrochloride and DMSO were added to LB liquid medium containing *P. syringae* (A) and *X. campestris* pv. *vesicatoria* (B), followed by 24 h of incubation, after which the OD_600_ values were measured. (C,D) Growth curve of *P. syringae* (C) and *X. campestris* pv. *vesicatoria* (D) in LB liquid medium supplemented with various concentrations of gatifloxacin hydrochloride or DMSO. The OD_600_ was measured every 3 h starting from 12 h or 24 h post-incubation. The data are presented as mean ± standard deviation (*n* = 3). Different lowercase letters indicate statistical significance (one-way ANOVA, *p* < 0.05).

**Figure 7:**
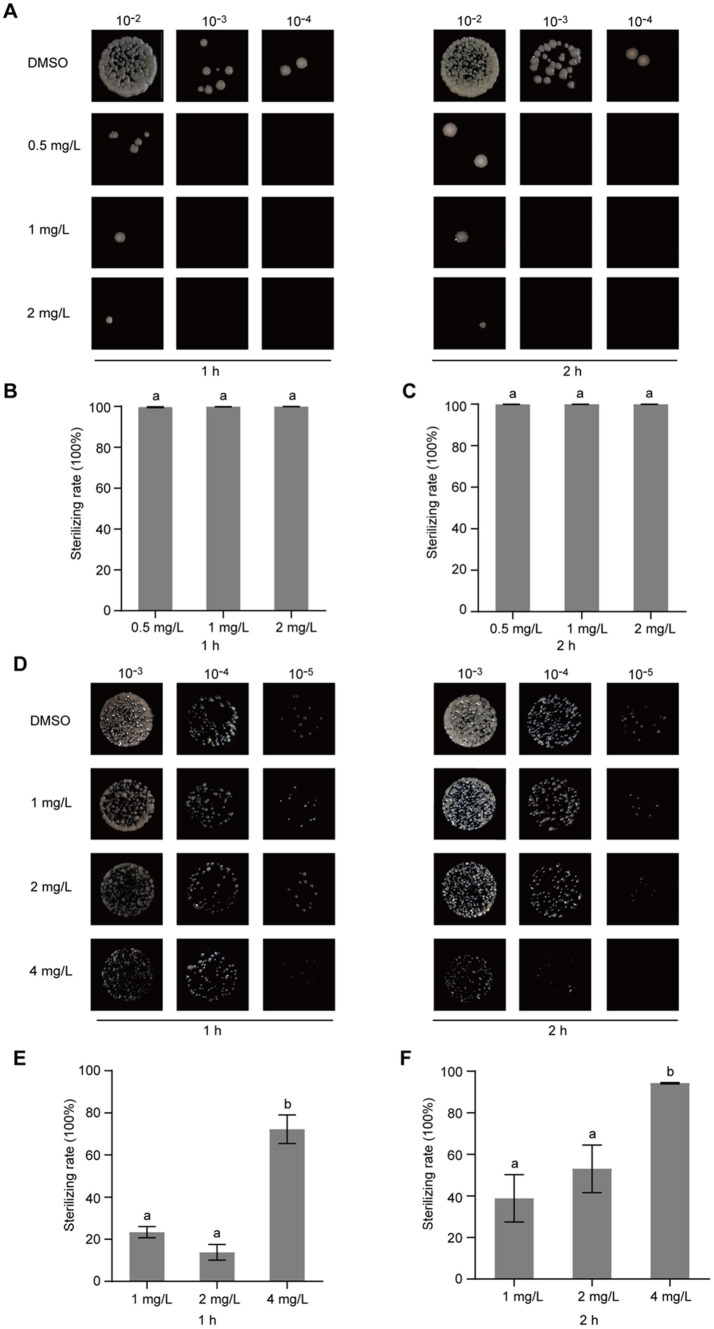
Gatifloxacin hydrochloride exhibits bactericidal activity against *P. syringae* and *X. campestris* pv. *vesicatoria*. Different concentrations of gatifloxacin hydrochloride and DMSO were added to a suspension of *P. syringae* (A–C) and *X. campestris* pv. *vesicatoria* (D–F) at an OD_600_ of 0.1, followed by co-incubation of the mixture at 28°C. After treatment for either 1 h or 2 h, the mixture was harvested, diluted, and plated onto the LB agar plates. (A,D) Colony shape of *P. syringae* (A) and *X. campestris* pv. *vesicatoria* (D) after gatifloxacin hydrochloride or DMSO treatment. (B,C,E,F) The number of surviving *P. syringae* (B,C) and *X. campestris* pv. *vesicatoria* (E,F) in the 10^−4^ dilution was quantified, using the bacterial count from the DMSO treatment group as a control to calculate the bactericidal rate. The data are presented as mean ± standard deviation (*n* = 3). Different lowercase letters indicate statistical significance (one-way ANOVA, *p* < 0.05).

The identification of broad-spectrum antibacterial agents is pivotal for the management of plant diseases. To ascertain the broad-spectrum antibacterial potential of gatifloxacin hydrochloride, we initially assessed its inhibitory impact on *P. syringae*, a prominent bacterial pathogen based on its academic significance and economic impact (Mansfield et al., 2012). At a concentration of 0.015 mg/L, gatifloxacin hydrochloride induced only a marginal inhibition of *P. syringae* growth, whereas a concentration of 0.5 mg/L nearly completely arrested its growth (Figure 6A). Subsequently, we evaluated the inhibitory effect of gatifloxacin hydrochloride on *X. campestris* pv. *vesicatoria*. Consistent with the results observed for *P. syringae*, 0.5 mg/L of gatifloxacin hydrochloride substantially inhibited the growth of *X. campestris* pv. *vesicatoria* in liquid culture (Figure 6B). Then, we performed a growth curve for both *P. syringae* and *X. campestris* pv. *vesicatoria* in the absence or presence of gatifloxacin hydrochloride. Supplement with different concentrations of gatifloxacin hydrochloride significantly suppressed the growth of *P. syringae* and *X. campestris* pv. *vesicatoria* in a liquid medium in a 24-h time interval (Figures 6C,D). We further tested the bactericidal activity of gatifloxacin hydrochloride against *P. syringae* and *X. campestris* pv. *vesicatoria*. Compared to the DMSO control, treatment with 1 mg/L of gatifloxacin hydrochloride killed 98% of the *P. syringae,* and 4 mg/L of gatifloxacin hydrochloride killed approximately 90% of the *X. campestris* pv. *vesicatoria* (Figure 7), indicating a strong bactericidal activity against both pathogens. These findings suggest that gatifloxacin hydrochloride possesses broad-spectrum antibacterial activity against plant pathogenic bacteria.

**Figure 8:**
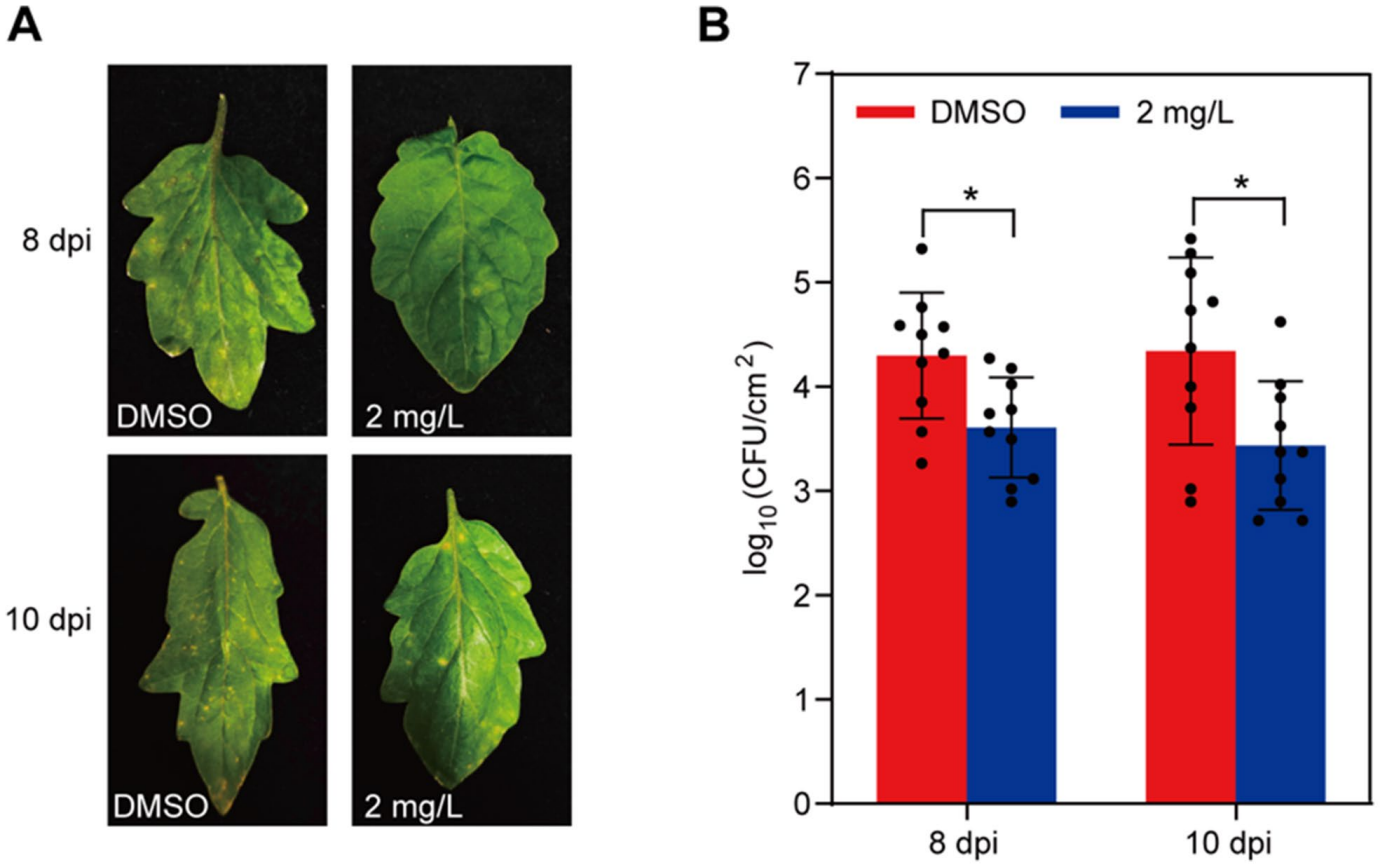
Gatifloxacin hydrochloride treatment delays disease development by *P. syringae*. (A) Four-week-old tomato leaves were sprayed with either DMSO or 2 mg/L of gatifloxacin hydrochloride, followed by inoculating with *P. syringae*. Disease symptoms were photographed 8 and 10 days post-inoculation (dpi). (B) Bacterial colonies of *P. syringae* were quantified after 8 or 10 days post-inoculation. Data are presented as mean ± standard deviation (*n* = 10). *p < 0.05 (Student’s *t*-test).

To elucidate whether gatifloxacin hydrochloride could impede disease progression caused by *P. syringae*, we inoculated tomato leaves with *P. syringae* and subsequently treated them with 2 mg/L of gatifloxacin hydrochloride. In comparison with the DMSO control, the development of bacterial spots was significantly slower in the gatifloxacin hydrochloride-treated leaves at 8 and 10 days post-inoculation (Figure 8A). In addition, we quantified the bacterial load in the inoculated tomato leaves and found that treatment with gatifloxacin hydrochloride markedly reduced bacterial titers (Figure 8B). In summary, these results collectively indicate that gatifloxacin hydrochloride exhibits broad-spectrum antibacterial activity against plant pathogenic bacteria and holds promise as a potential agent for the control of bacterial diseases in plants.

## Discussion

In this study, the antibacterial activity of gatifloxacin hydrochloride was first evident through its ability to inhibit the growth of *R. solanacearum* in a concentration-dependent manner. In particular, at a concentration as low as 0.125 mg/L, gatifloxacin hydrochloride demonstrated significant inhibitory effects, aligning with previous studies that have shown the efficacy of fluoroquinolones against a variety of bacterial pathogens (Maris et al., 2021). The bactericidal activity of gatifloxacin hydrochloride was further confirmed through its ability to reduce bacterial viability by over 89% at 0.25 mg/L after a 1-h treatment. This rapid bactericidal action is crucial for disease management as it suggests that gatifloxacin hydrochloride could be effective in controlling the early stages of infection. Several plant-derived compounds have been demonstrated to have good antibacterial activity against *R. solanacearum* (Pradhanang et al., 2003; Yang et al., 2016, 2021; Han et al., 2021; Li et al., 2021; Xia et al., 2023); however, their MIC values are much higher than gatifloxacin hydrochloride. For example, 7-methoxycoumarin with concentrations from 25 mg/L to 100 mg/L significantly suppressed the growth of *R. solanacearum* (Han et al., 2021), while in the case of gatifloxacin hydrochloride, 0.25 mg/L already completely inhibited the growth of *R. solanacearum* in liquid culture (Figure 2). Several other hydroxycoumarins, such as daphnetin, esculetin, and umbelliferone, suppressed the growth of *R. solanacearum* with concentrations ranging from 64 to 256 mg/L (Yang et al., 2016), which is approximately 300 times higher than the MIC of gatifloxacin hydrochloride. Caffeic acid also shows great potential in inhibiting *R. solanacearum* growth; however, the MIC of caffeic acid is 200 mg/L against *R. solanacearum* (Li et al., 2021). Thus, compared to reported natural products, gatifloxacin hydrochloride shows the best antibacterial activity against *R. solanacearum*. From the dosage perspective, using gatifloxacin hydrochloride has a substantial advantage as a very low concentration of the compound can inhibit *R. solanacearum* growth and delay bacterial wilt disease development. The broad-spectrum antibacterial activity of gatifloxacin hydrochloride was further demonstrated against *P. syringae* and *X. campestris* pv*. vesicatoria*, two economically important plant pathogens (Mansfield et al., 2012). The inhibitory effect on these pathogens suggests that gatifloxacin hydrochloride could be a valuable tool in integrated disease management strategies, particularly in crops where multiple bacterial pathogens are a concern.

Gatifloxacin hydrochloride is recognized for its efficacy in treating a variety of infections, including acute bacterial exacerbation of chronic bronchitis, acute sinusitis, community-acquired pneumonia, pyelonephritis, and gonorrhea (Keam et al., 2005). The primary mechanism by which gatifloxacin hydrochloride suppresses bacterial growth is the inhibition of bacterial DNA replication through interference with DNA gyrase and topoisomerase IV, enzymes essential for bacterial cell division (Keam et al., 2005; Rafii et al., 2005). Given its broad-spectrum antibacterial activity against both human and plant pathogenic bacteria, it is probable that gatifloxacin hydrochloride also targets DNA gyrase and topoisomerase IV in phytopathogenic bacteria such as *R. solanacearum* and *P. syringae*. As a small molecule compound, gatifloxacin hydrochloride is relatively safe and environmentally friendly. However, due to its antibacterial activity against human pathogens, caution is required when using it for plant disease control. Residues of gatifloxacin hydrochloride must be minimized to reduce potential side effects on humans. Despite this, the MIC of gatifloxacin hydrochloride against plant pathogenic bacterial (e.g., *R. solanacearum*) is significantly lower than other plant-derived compounds (Yang et al., 2016; Han et al., 2021; Li et al., 2021; Xia et al., 2023), suggesting its considerable potential as an alternative for controlling plant diseases in the agricultural system.

Biofilms are known to play a significant role in the pathogenesis of bacterial diseases by enhancing bacterial resistance to environmental stresses and antimicrobial agents (Yao and Allen, 2007; O’Loughlin et al., 2013; Shree et al., 2023). Our results showed that gatifloxacin hydrochloride significantly reduced biofilm formation by *R. solanacearum*, even at the lowest tested concentration of 0.0625 mg/L. This effect is particularly important given that biofilm-associated bacteria are often more resistant to conventional treatments (Shree et al., 2023). The ability of gatifloxacin hydrochloride to disrupt biofilm formation may contribute to its overall efficacy in managing bacterial diseases.

Our pot experiment with tomato plants confirmed the potential of gatifloxacin hydrochloride to delay disease development caused by *R. solanacearum*. The reduction in disease severity and the delay in symptom onset following gatifloxacin hydrochloride treatment highlight its potential as a prophylactic or therapeutic agent in agricultural settings. This aligns with the growing interest in using natural compounds as alternatives to synthetic pesticides, which are often associated with environmental and resistance concerns (Tang et al., 2021; Ayilara et al., 2023). The use of natural compounds such as gatifloxacin hydrochloride offers an environmentally friendly approach to disease control. However, it is crucial to consider the potential for the development of resistance among bacterial populations. Continued monitoring and research will be necessary to understand the long-term effectiveness and resistance dynamics associated with the use of gatifloxacin hydrochloride.

In summary, this investigation has established that gatifloxacin hydrochloride exhibits robust antimicrobial activity against various plant pathogenic bacteria such as *R. solanacearum*, *P. syringae*, and *X. campestris* pv. *vesicatoria*. The results highlight its efficacy not only in inhibiting bacterial proliferation but also in diminishing biofilm production and retarding the onset of disease development, thereby suggesting its utility as an effective tool in agricultural pest management. As gatifloxacin hydrochloride is a small-molecule compound, these discoveries reinforce the notion that small-molecule compounds hold significant promise as versatile antibacterials for combating plant-based infections. Future research should focus on optimizing its application, understanding its mechanisms of action, and assessing its potential for resistance development.

## Data Availability

The original contributions presented in the study are included in the article/Supplementary material, further inquiries can be directed to the corresponding authors.
