# Corrigendum: Gatifloxacin hydrochloride confers broad-spectrum antibacterial activity against phytopathogenic bacteria

**DOI:** 10.3389/fmicb.2025.1618021

**Published:** 2025-05-14

**Authors:** Yanxia Huang, Bin Peng, Chenhui Li, Yuqin Wu, Zixian Zeng, Moh Tariq, Lin Jiang, Shun-xiang Li, Dousheng Wu

**Affiliations:** ^1^Hunan Key Laboratory of Plant Functional Genomics and Developmental Regulation, College of Biology, Hunan University, Changsha, China; ^2^Hunan Engineering Technology Research Center for Bioactive Substance Discovery of Chinese Medicine, School of Pharmacy, Hunan University of Chinese Medicine, Changsha, China

**Keywords:** gatifloxacin hydrochloride, antibacterial, *Ralstonia solanacearum*, *Pseudomonas syringae*, *Xanthomonas campestris* pv. Vesicatoria

In the published article, there was an error in the legend for **Figure 1**, page 4 as published. The compound library composition was inaccurately described as exclusively containing 58 microorganism-derived natural products, whereas it included 57 microorganism-derived small molecules and 1 fully synthetic compound. The corrected legend appears below.

“Screening of microorganism-derived and synthetic compounds against *R. solanacearum*. The effects of 57 microorganism-derived small molecules and 1 fully synthetic compound on the growth of *R. solanacearum* were investigated.”

In the published article, there was an error. The source of the fully synthetic compounds was incorrectly described as “microorganism-derived compounds” in the text.

A correction has been made to **Abstract Section**, page 1, paragraph 1. This sentence previously stated:

“In this study, we screened a library of 58 microorganism-derived natural products for their antibacterial activity against *R. solanacearum*.”

The corrected sentence appears below:

“In this study, we screened a library of 57 microorganism-derived small molecule compounds and 1 fully synthetic small molecule compound for their antibacterial activity against *R. solanacearum*.”

A correction has been made to **Abstract Section**, page 1, paragraph 1. This sentence previously stated:

“In summary, our results demonstrate the great potential of microorganism-derived compounds as broad-spectrum antibacterial compounds, providing alternative ways for the efficient control of bacterial plant diseases.”

The corrected sentence appears below:

“In summary, our results demonstrate the great potential of microorganism-derived and synthetic small molecules as broad-spectrum antibacterial compounds, providing alternative ways for the efficient control of bacterial plant diseases.”

A correction has been made to **Introduction Section**, page 2, paragraph 5. This sentence previously stated:

“Gatifloxacin hydrochloride is a microorganism-derived compound with promising antibacterial activities against both Gram positive and Gram-negative bacteria, including some anaerobic organisms and mycobacteria.”

The corrected sentence appears below:

“Gatifloxacin hydrochloride is a fully synthetic small molecule with promising antibacterial activities against both Gram positive and Gram-negative bacteria, including some anaerobic organisms and mycobacteria.”

A correction has been made to **Introduction Section**, page 2, paragraph 6. This sentence previously stated:

“In this study, we screened a microorganism-derived compound library to identify compounds that show good antibacterial activity against *R. solanacearum*.”

The corrected sentence appears below:

“In this study, we screened a compound library comprising 57 microorganism-derived compounds and 1 fully synthetic compound to identify compounds that show good antibacterial activity against *R. solanacearum*.”

A correction has been made to **Materials and methods Section**, *Microorganism-derived compounds information*, page 2. This heading previously stated:

“Microorganism-derived compounds information.”

The corrected heading appears below:

“Small molecule compounds information.”

A correction has been made to **Materials and methods Section**, *Microorganism-derived compounds information*, page 2, paragraph 1. This sentence previously stated:

“In this study, all microorganism-derived compounds (HPLC purity >99%) were acquired from TargetMol.”

The corrected sentence appears below:

“In this study, all small molecule compounds (HPLC purity >99%) were acquired from TargetMol.”

A correction has been made to **Results Section**, *A screen identifies gatifloxacin hydrochloride as a novel antibacterial agent against Ralstonia solanacearum*, page 4, paragraph 1. This sentence previously stated:

“To further identify natural compounds that can inhibit *R. solanacearum* growth, we screened a library consisting of 58 microorganism-derived compounds and tested their antibacterial activity against *R. solanacearum* at a concentration of 10 μM.”

The corrected sentence appears below:

“To further identify natural compounds that can inhibit *R. solanacearum* growth, we screened a library consisting of 57 microorganism-derived small molecule compounds and 1 fully synthetic small molecule compound and tested their antibacterial activity against *R. solanacearum* at a concentration of 10 μM.”

A correction has been made to **Results Section**, *A screen identifies gatifloxacin hydrochloride as a novel antibacterial agent against Ralstonia solanacearum*, page 4, paragraph 1. This sentence previously stated:

“In summary, the screening identified several microorganism-derived natural compounds as novel antibacterial agents against *R. solanacearum*.”

The corrected sentence appears below:

“In summary, the screening identified several small molecule compounds as novel antibacterial agents against *R. solanacearum*.”

A correction has been made to **Results Section**, *Gatifloxacin hydrochloride delays bacterial wilt disease development*, page 5, paragraph 1. This sentence previously stated:

“Given that gatifloxacin hydrochloride shows strong antibacterial activity against *R. solanacearum* and reduces biofilm formation, we hypothesized that this microorganism-derived compound could delay bacterial wilt disease development caused by this pathogen. *R. solanacearum* has a wide range of host plants, with Solanaceae plants, particularly tomato, being the most common hosts.”

The corrected sentence appears below:

“Given that gatifloxacin hydrochloride shows strong antibacterial activity against *R. solanacearum* and reduces biofilm formation, we hypothesized that this small molecule compound could delay bacterial wilt disease development caused by this pathogen. *R. solanacearum* has a wide range of host plants, with Solanaceae plants, particularly tomato, being the most common hosts.”

A correction has been made to **Discussion Section**, page 8, paragraph 2. This sentence previously stated:

“As a microorganism-derived natural compound, gatifloxacin hydrochloride is relatively safe and environmentally friendly.”

The corrected sentence appears below:

“As a small molecule compound, gatifloxacin hydrochloride is relatively safe and environmentally friendly.”

A correction has been made to **Discussion Section**, paragraph 5. This sentence previously stated:

“As gatifloxacin hydrochloride originates from microbial sources, these discoveries reinforce the notion that compounds derived from microorganisms hold significant promise as a versatile antibacterial for combating plant-based infections.”

The corrected sentence appears below:

“As gatifloxacin hydrochloride is a small-molecule compound, these discoveries reinforce the notion that small-molecule compounds hold significant promise as versatile antibacterials for combating plant-based infections.”

The authors apologize for this error and state that this does not change the scientific conclusions of the article in any way. The original article has been updated.

